# Supplementary material for: Frequency of Restaurant, Delivery and Takeaway Usage Is Not Related to BMI among Adults in Scotland
Source: Nutrients. 2020 Aug 19;12(9):2501. doi: 10.3390/nu12092501 (PMC7551913; doi:10.3390/nu12092501)
Supplement: Supplementary file 1 [file nutrients-12-02501-s001.pdf]

Table S1: Socio-demographic survey

1. **Please, write down Your mobile phone number (we need your mobile number to register you in our Prize Draw, also to send to you a reminder for our next survey):**
2. **Please enter your age:**
3. **Please write your postcode district (the first two letters and two numbers, example AB10):**
4. **What is your sex?**
5. **If female:**
   Are you pregnant or breastfeeding?
6. **Please write your height:**
7. **Please write your weight:**
8. **Which race/ethnicity best describes you? (Please choose only one.):**
   a. White
   b. Asian
   c. Black
   d. Mixed
9. **Are you currently employed?**
   a. **Yes**
   b. **No**
   c. **Student**
10. **How many people currently live in your household?**
11. **How many children live in your household (aged 17 or younger only)?**
12. **How would you describe your dietary habits?**
    a. Regular diet (no restrictions)
    b. Vegetarian (you only eat grains and pulses, vegetables, fruits, milk and milk products and eggs)
    c. As above but you avoid eggs
    d. As above but you avoid milk and eggs
    e. As above but you avoid milk, eggs and grain products
    f. Fruitarian (you only eat fruits and fruit products)
    g. Pescatarian (you only eat vegetables, fruits, fish, milk and milk products and eggs)
    h. Other:

13. Do you have any food allergy or intolerance?

a. **Yes**

b. **No**

14. Do you remember how many steps you did yesterday?

Table S2: Food Outlet Usage survey

Do you wish to continue with the survey?

Yes □ No □

Did you go to any restaurants or used any kind of food services such as ordering a food delivery or takeaway today?

- Yes
- No

If yes, please, which one of these have been used:
Tick all that apply

Type of restaurant	Breakfast	Lunch	Dinner	Snack	None for today
Sit down restaurant where you have to pay after you finish eating	□	□	□	□	□
A fast food restaurant or a café where you pay at the counter before eating	□	□	□	□	□
Food delivery where it is ordered and delivered to your home	□	□	□	□	□

Food takeaway where you order and take it out from the food establishment	☐	☐	☐	☐	☐

Table S3: ANOVA analysis: unadjusted BMI vs the frequency of food outlet usage for 7-consecutive days segregated by sex.

Males						
**Food outlet type Number of usages**	**N**	**Mean unadjusted BMI**	**SD**	**99% CI**	**Bonferroni P-value**	**Figure 3**
**FSR**					**P=0.12**	**A**
0.00	121	27.625	4.372	(26.691; 28.560)		
1.00	83	26.843	3.588	(25.715; 27.972)		
2.00	57	26.244	3.598	(24.882; 27.606)		
3.00	19	27.264	3.807	(24.905; 29.623)		
4.00	8	29.46	4.31	(25.82; 33.09)		
5.00	1	32.92	*	(22.64; 43.20)		
6.00	2	27.265	0.769	(19.994; 34.536)		
**FFR**					**P=0.13**	**B**
0.00	93	26.629	3.458	(25.563; 27.695)		
1.00	93	27.730	4.427	(26.664; 28.796)		
2.00	54	27.482	3.801	(26.084; 28.881)		
3.00	33	26.006	3.855	(24.217; 27.796)		
4.00	12	28.73	4.63	(25.76; 31.70)		
5.00	2	27.47	3.50	(20.20; 34.73)		
6.00	3	29.44	5.18	(23.50; 35.37)		
8.00	1	22.03	*	(11.75; 32.31)		
**Delivery**					**P=0.96**	**C**
0.00	171	27.177	3.732	(26.380; 27.973)		
1.00	68	27.418	4.595	(26.155; 28.681)		
2.00	42	26.856	4.419	(25.249; 28.463)		
3.00	8	26.856	1.894	(23.174; 30.539)		
4.00	2	26.66	2.36	(19.29; 34.02)		
**Takeaways**					**P=0.40**	**D**
0.00	173	27.260	3.969	(26.473; 28.047)		
1.00	89	27.298	4.242	(26.201; 28.396)		
2.00	25	25.867	2.815	(23.797; 27.938)		
3.00	3	28.10	6.14	(22.12; 34.08)		
4.00	1	31.22	*	(20.87; 41.57)		
**TFO**					**P=0.64**	**E**
0.00	40	26.728	3.345	(25.085; 28.371)		
1.00	39	27.585	4.238	(25.921; 29.249)		
2.00	28	27.892	4.330	(25.929; 29.856)		
3.00	39	27.003	4.137	(25.339; 28.667)		

4.00	41	27.361	4.198	(25.738; 28.984)		
5.00	31	26.255	4.029	(24.388; 28.121)		
6.00	36	27.856	4.001	(26.125; 29.588)		
7.00	20	26.167	3.803	(23.844; 28.490)		
8.00	17	27.458	3.748	(24.938; 29.979)		
**Females**						
	**N**	**Mean unadjusted BMI**	**SD**	**99% CI**	**Bonferroni P-value**	**Figure 4**
**FSR**					**P=0.04**	**A**
0.00	185	25.058	3.872	(24.275; 25.840)		
1.00	122	25.626	4.301	(24.662; 26.589)		
2.00	54	25.963	4.266	(24.514; 27.411)		
3.00	23	27.750	4.716	(25.531; 29.969)		
4.00	5	23.12	3.11	(18.36; 27.88)		
5.00	1	23.71	*	(13.07; 34.35)		
**FFR**					**P=0.02**	**B**
0.00	165	25.117	3.986	(24.291; 25.943)		
1.00	133	25.861	4.329	(24.941; 26.781)		
2.00	44	25.328	2.766	(23.728; 26.928)		
3.00	34	25.083	4.576	(23.262; 26.903)		
4.00	11	29.36	5.81	(26.16; 32.56)		
5.00	2	23.924	0.531	(16.419; 31.428)		
8.00	1	19.80	*	(9.19; 30.41)		
**Delivery**					**P=0.09**	**C**
0.00	250	25.172	3.973	(24.497; 25.848)		
1.00	92	25.633	3.800	(24.519; 26.746)		
2.00	39	26.757	5.332	(25.047; 28.467)		
3.00	5	26.44	5.64	(21.67; 31.22)		
4.00	4	28.67	5.78	(23.33; 34.01)		
**Takeaways**					**P=0.32**	**D**
0.00	253	25.376	4.138	(24.702; 26.050)		
1.00	94	25.618	3.997	(24.512; 26.724)		
2.00	32	25.542	3.766	(23.647; 27.437)		
3.00	6	29.13	8.21	(24.75; 33.51)		
4.00	4	24.893	1.722	(19.532; 30.253)		
6.00	1	21.66	*	(10.94; 32.38)		
**TFO**					**P=0.41**	**E**
0.00	81	24.678	4.055	(23.485; 25.870)		
1.00	53	25.552	4.456	(24.078; 27.026)		
2.00	62	25.453	3.425	(24.090; 26.816)		

3.00	45	25.118	3.521	(23.519; 26.718)	
4.00	49	25.567	3.555	(24.034; 27.100)	
5.00	43	25.763	4.010	(24.127; 27.399)	
6.00	26	26.274	4.101	(24.169; 28.378)	
7.00	20	26.63	6.42	(24.23; 29.03)	
8.00	10	27.42	6.94	(24.03; 30.81)	
9.00	1	29.56	*	(18.82; 40.29)	

N= number of participants in each group, FSR= Full-Service Restaurants; FFR= Fast Food Restaurants; BMI= Body Mass Index; SD= Standard Deviation, CI= Confidence Interval. Results of the ANOVA are shown. Significance is where P< 0.01 (after Bonferroni correction)

Table S4: ANOVA analysis: Adjusted mean BMI vs the frequency of food outlet usage for 7-consecutive days

Males						
**Food outlet type Number of usages**	**N**	**Mean adjusted BMI**	**SD**	**99% CI**	**Bonferroni P-value**	**Figure 5**
**FSR**					**P=0.13**	**A**
0.00	121	26.720	4.171	(25.828, 27.613)		
1.00	83	25.854	3.360	(24.777, 26.932)		
2.00	57	25.372	3.610	(24.072, 26.672)		
3.00	19	25.976	3.682	(23.724, 28.227)		
4.00	8	28.04	3.34	(24.57, 31.51)		
5.00	1	31.82	*	(22.00, 41.63)		
6.00	2	26.06	1.51	(19.12, 33.00)		
**FFR**					**P=0.16**	**B**
0.00	93	25.624	3.150	(24.606, 26.643)		
1.00	93	26.553	4.216	(25.534, 27.571)		
2.00	54	26.623	3.854	(25.287, 27.960)		
3.00	33	25.234	3.712	(23.524, 26.943)		
4.00	12	28.22	4.32	(25.38, 31.05)		
5.00	2	26.63	3.80	(19.69, 33.58)		
6.00	3	28.73	5.60	(23.06, 34.40)		
8.00	1	26.21	*	(16.39, 36.03)		
**Delivery**					**P=0.90**	**C**
0.00	171	26.162	3.487	(25.467, 26.856)		
1.00	68	26.435	4.522	(24.981, 27.888)		
2.00	42	26.192	4.246	(24.422, 27.962)		
3.00	8	25.633	1.780	(23.430, 27.836)		
4.00	2	25.40	1.96	(-62.64, 113.43)		
**Takeaways**					**P=0.62**	**D**
0.00	173	26.296	3.672	(25.543, 27.049)		
1.00	89	26.275	4.282	(25.225, 27.324)		
2.00	25	25.151	2.678	(23.171, 27.132)		
3.00	3	27.61	5.80	(21.89, 33.33)		
4.00	1	27.81	*	(17.91, 37.71)		
**TFO**					**P=0.56**	**E**
0.00	40	25.621	2.973	(24.348, 26.894)		
1.00	39	26.516	3.806	(24.864, 28.169)		
2.00	28	26.790	4.230	(24.575, 29.004)		
3.00	39	26.216	4.210	(24.388, 28.044)		
4.00	41	26.299	4.153	(24.545, 28.053)		

5.00	31	25.308	3.717	(23.472, 27.144)		
6.00	36	26.979	3.758	(25.273, 28.685)		
7.00	20	25.341	3.661	(22.999, 27.683)		
8.00	17	26.748	3.631	(24.176, 29.321)		
**Females**						
	**N**	**Mean adjusted BMI**	**SD**	**99% CI**	**Bonferroni P-value**	**Figure 6**
**FSR**					**P=0.12**	**A**
0.00	185	25.919	3.845	(25.145, 26.692)		
1.00	122	26.254	4.163	(25.302, 27.207)		
2.00	54	26.664	4.398	(25.232, 28.095)		
3.00	23	27.949	4.644	(25.755, 30.143)		
4.00	5	23.35	2.50	(18.65, 28.06)		
5.00	1	23.59	*	(13.07, 34.11)		
**FFR**					**P=0.03**	**B**
0.00	165	25.843	3.938	(25.028, 26.658)		
1.00	133	26.531	4.247	(25.623, 27.438)		
2.00	44	25.974	2.589	(24.395, 27.552)		
3.00	34	25.995	4.596	(24.200, 27.790)		
4.00	11	30.02	5.94	(26.87, 33.18)		
5.00	2	24.924	0.485	(17.523, 32.326)		
8.00	1	21.61	*	(11.15, 32.08)		
**Delivery**					**P=0.21**	**C**
0.00	250	25.819	3.882	(25.181, 26.456)		
1.00	92	26.384	3.694	(25.371, 27.397)		
2.00	39	27.722	5.272	(25.433, 30.011)		
3.00	5	27.48	5.74	(15.66, 39.29)		
4.00	4	30.11	5.88	(12.94, 47.29)		
**Takeaways**					**P=0.12**	**D**
0.00	253	26.128	4.072	(25.464, 26.791)		
1.00	94	26.202	3.992	(25.114, 27.291)		
2.00	32	26.311	3.551	(24.445, 28.177)		
3.00	6	30.03	7.99	(25.72, 34.33)		
4.00	4	25.74	2.05	(20.46, 31.01)		
6.00	1	22.61	*	(12.06, 33.17)		
**TFO**					**P=0.42**	**E**
0.00	81	25.579	4.140	(24.687, 26.471)		
1.00	53	26.038	4.178	(24.935, 27.140)		
2.00	62	26.209	3.294	(25.190, 27.229)		
3.00	45	25.713	3.298	(24.516, 26.909)		
4.00	49	26.098	3.578	(24.951, 27.245)		

5.00	43	26.642	4.133	(25.417, 27.866)	
6.00	26	26.954	3.894	(25.379, 28.528)	
7.00	20	27.48	6.43	(25.69, 29.28)	
8.00	10	28.37	6.81	(25.84, 30.91)	
9.00	1	28.54	*	(20.52, 36.57)	
**N= number of participants in each group, FSR= Full-Service Restaurants; FFR= Fast Food Restaurants; BMI= Body Mass Index; SD= Standard Deviation, CI= Confidence Interval. Results of the ANOVA are shown. Significance is where P< 0.01 (after Bonferroni correction)**					
